# Identification of prognostic factors associated with contemporary lifestyles in community-dwelling young stroke survivors

**DOI:** 10.3389/fmed.2026.1928635

**Published:** 2026-09-23

**Authors:** Yingyi Dai, Shasha Lei, Dongshi Li, Xuying He, Yicheng Liang

**Affiliations:** 1 Department of Neurology, The Affiliated Guangdong Second Provincial General Hospital of Jinan University, Guangzhou, Guangdong, China; 2 Department of Neurology, The Second People's Hospital of Panyu Guangzhou, Guangzhou, Guangdong, China

**Keywords:** contemporary lifestyles, population study, prognostic factors, stroke, young adults

## Abstract

**Background:**

Over the past few decades, the global incidence of stroke among young adults (aged 18–50 years) has risen significantly. Given their prolonged post-stroke life expectancy, this trend has resulted in a substantial burden of long-term disability. Beyond traditional vascular prognostic factors, unhealthy modern lifestyles are increasingly contributing to this trend.

**Method:**

This study enrolled community-dwelling adults from three communities in Guangzhou, who were stratified into a young adult group (aged 18–49 years) and an older adult group (aged ≥50 years). Baseline questionnaires were administered to collect demographic characteristics, educational attainment, occupation, medical and family histories, lifestyle factors, and the Essen Stroke Risk Score (ESRS). During a one-year telephone follow-up, we assessed the recurrence of stroke, modified Rankin Scale (mRS) scores, and changes in the ESRS from baseline.

**Result:**

Young adults exhibited higher educational attainment and a distinct occupational distribution. The older adult cohort demonstrated a higher prevalence of preexisting medical conditions, as well as a more frequent family history of coronary heart disease and cancer. Young participants reported engaging in less physical exercise, domestic chores, and social interaction; however, they reported better sleep quality and a higher rate of cohabitation. In the unadjusted analysis, the young adult group exhibited a lower proportion of favorable functional outcomes (mRS 0–1: 60% vs. 65%), with an unadjusted odds ratio (OR) of 0.789 [95% confidence interval (CI): 0.670-0.930]. However, this disparity was attenuated and became non-significant following adjustment, the OR was 0.904 (95% CI: 0.730–1.120, *p* = 0.355). Ischemic stroke recurrence rates were comparable between the two groups (4% vs. 6%), whereas the older group exhibited a more pronounced change in ESRS scores.

**Conclusion:**

The initially observed disparity in functional outcomes between young and older stroke patients appears to be heavily influenced by distinct, modifiable lifestyle factors. Early identification of and timely intervention against these prognostic factors are paramount for reducing the recurrence of stroke among young adults and minimizing its long-term sequelae.

## 1 Introduction

Stroke in young adults has increasingly emerged as a critical focal point in both clinical medicine and public health. Mounting epidemiological evidence indicates a concerning demographic shift, with the incidence of stroke progressively skewing toward younger populations. Over the past few decades, the global incidence of stroke among young adults has exhibited a steady upward trajectory. In Western nations, stroke incidence in individuals aged 20–64 years has surged by approximately 25%. Similarly, low- and middle-income countries are witnessing a demographic transition in stroke burden; for instance, nearly 12% of stroke cases in India now occur in individuals under 40 years of age (1). On a global scale, the 15–39-year age cohort has recorded the highest relative increase in ischemic stroke incidence across all age groups (2).

Stroke exerts a profound impact on patients’ quality of life, with particularly severe consequences for the young adult population. This demographic often faces a heavy socioeconomic burden due to long-term disability and decades of lost labor productivity (3, 4). Although stroke recurrence can further exacerbate these adverse outcomes, a considerable proportion of young stroke patients remain inadequately aware of recurrence-associated risk factors and secondary prevention measures. Therefore, standardized daily life management and rigorous risk factor control are crucial for preventing stroke recurrence in young patients. Young adulthood is a critical stage for the formation of independent lifestyle habits, representing a vital window of opportunity for implementing targeted preventive interventions.

Driven by rapid societal modernization, young adults are increasingly adopting Westernized lifestyles characterized by sedentary behavior, smoking, excessive alcohol consumption, and diets high in fat and cholesterol. Furthermore, the persistent rise in the prevalence of human immunodeficiency virus (HIV) infection and acquired immunodeficiency syndrome (AIDS) has amplified the stroke risk, compounding the disease burden in this demographic (5, 6). Notably, emerging evidence demonstrates that traditional vascular risk factors previously confined to older populations—such as hypertension and diabetes mellitus—have now become major precipitants of stroke in young adults (7–9). Concurrently, the escalating prevalence of obesity among the youth acts as a primary driver exacerbating this growing disease burden (10–12).

Therefore, implementing comprehensive risk factor assessments and optimizing secondary prevention strategies in young stroke patients are critical for reducing future vascular events and improving long-term functional outcomes. In this context, the present study systematically evaluated recurrence-associated risk factors among community-dwelling young stroke patients in the clinically stable phase. This study aims to provide clinicians with the evidence base required to precisely identify individuals at high risk of recurrence, thereby facilitating the development of individualized secondary prevention regimens. Such strategies should encompass comprehensive measures, including enhancing awareness of stroke recurrence risks and promoting healthy behavioral interventions (e.g., dietary modification, regular physical activity, and smoking cessation). Ultimately, our findings aim to inform clinical practice and optimize existing secondary prevention pathways, with the goal of effectively reducing stroke recurrence rates in the young adult population.

## 2 Materials and methods

This study was conducted in accordance with the principles of the World Medical Association's Declaration of Helsinki. The clinical data analysis complied with the Ethics Committee policies of The Affiliated Guangdong Second Provincial General Hospital of Jinan University [Approval number: (2021-KZ-027-04)].

### 2.1 Study participants

Three communities with relatively stable populations (each with approximately 10,000 or more permanent residents) in the old urban area of Guangzhou were selected as the study sites. Potential participants were initially identified using pre-existing chronic disease management registries maintained by the local Community Health Centers. Following initial contact, interested individuals were comprehensively screened, and eligible patients were enrolled on a strictly voluntary basis after providing written informed consent. To ensure high data quality and participant retention, the baseline assessments and subsequent one-year follow-ups utilized highly structured questionnaires predominantly featuring multiple-choice options for ease of administration. Crucially, the entire identification, enrollment, and follow-up processes were conducted by a single, dedicated team of trained researchers to minimize inter-rater variability and maximize compliance.

The inclusion criteria were as follows: (1) aged 18–80 years; (2) formally registered permanent residents living long-term in the aforementioned communities; (3) having a confirmed history of ischemic stroke; and (4) currently in a clinically stable phase, defined as a modified Rankin Scale (mRS) score of ≤3 and no new vascular events within the 3 months prior to enrollment.

The exclusion criteria were: (1) patients in the acute phase of stroke (within 2 weeks of onset) or with clinically unstable conditions (e.g., progressive stroke, concurrent uncontrolled severe infection, or malignant hypertension); (2) individuals with other severe active neurological disorders (e.g., intracranial infection, uncontrolled epilepsy, progressive neurodegenerative diseases) or severe systemic diseases (e.g., end-stage malignancies, severe hepatic or renal dysfunction) that might impede follow-up assessments; and (3) those who refused to participate in the questionnaire survey or follow-up evaluations.

Based on age, the enrolled participants were stratified into a young adult group (18–49 years) and a middle-aged and older adult group (≥50 years).

### 2.2 Data acquisition

Trained investigators conducted face-to-face interviews using a standardized, structured questionnaire for all subjects meeting the inclusion and exclusion criteria. The questionnaire collected baseline demographic data, including sex, height, weight, and the frequency of annual physical examinations. Educational attainment was categorized as primary school or below, junior high school, vocational/senior high school, and bachelor's degree or above. Occupational categories included professionals/technicians, self-employed individuals, corporate managers, farmers, civil servants, students, blue-collar workers, unemployed individuals, active-duty military personnel, office clerks, freelancers, and others.

The questionnaire also evaluated lifestyle-related factors, encompassing personal medical history, family history of genetic diseases, smoking and alcohol consumption, frequency of physical exercise, frequency of domestic chores, weekly interpersonal interactions, sleep habits, sleep duration, and living environment. Additionally, all participants underwent a baseline assessment using the Essen Stroke Risk Score (ESRS). Personal medical history, including hypertension, heart disease, tumors, and other conditions, were defined strictly by a self-reported history of prior physician diagnosis obtained during structured interviews. Lifestyle factors were quantified using structured criteria: ‘insufficient exercise/light physical labor’ was strictly defined as exercising less than 3 times per week and less than 30 min per session. Sleep disorders were systematically evaluated across specific clinical phenotypes, including difficulty falling asleep, early awakening, difficulty returning to sleep, excessive dreaming, and abnormal overall sleep duration.

One year after study enrollment, telephone follow-ups were conducted to assess ischemic stroke recurrence and evaluate changes in the mRS and ESRS. The change in the ESRS was explicitly calculated by subtracting the ESRS at study enrollment from the ESRS assessed at the 1-year follow-up. This follow-up aimed to monitor longitudinal trends in the participants’ functional status and stroke risk profiles, as well as to analyze the associations between risk factors at study entry and one-year clinical outcomes.

### 2.3 Statistical analysis

Statistical analyses were performed using SPSS software, version 27.0. Missing data for participants lost to follow-up were addressed using multiple imputation under the missing at random (MAR) assumption. The imputation was performed using the Fully Conditional Specification method to generate 5 imputed datasets. Both baseline covariates and outcome variables were incorporated as predictors in the imputation model. Finally, the statistical results from these imputed datasets were pooled to generate the final estimates, allowing the final outcome analyses to be conducted on the full baseline cohort.

Descriptive analyses were utilized to summarize baseline characteristics. Continuous variables were expressed as the mean ± standard deviation (SD) or median (IQR), depending on the data distribution, which was evaluated using the Kolmogorov–Smirnov test. Categorical variables were presented as frequencies and percentages. Differences between the two age groups were compared using the Chi-square test for categorical variables, the independent samples t-test for normally distributed continuous variables, and the Mann–Whitney U test for non-normally distributed continuous data.

Functional outcomes and ischemic stroke recurrence at the one-year follow-up were evaluated using multivariable binary logistic regression models. Specifically, models were constructed to estimate the adjusted ORs for a favorable functional outcome (defined as mRS 0–1) and stroke recurrence. Conversely, to evaluate the change in the ESRS from baseline to the one-year follow-up—a multivariable generalized linear model was employed, with the effect estimates reported as adjusted *β* coefficients. In all multivariable models, potential confounders were strictly selected and adjusted for if they demonstrated a statistically significant difference (*P* < 0.05) in the univariate analyses. These comprehensively included demographics, medical and family history, contemporary lifestyle factors, sleep habits, and living arrangements. Results are presented as unadjusted and adjusted effect estimates (ORs or *β* coefficients) with their corresponding 95% CIs. All statistical tests were two-sided, and a two-tailed *P* value < 0.05 was considered statistically significant.

## 3 Result

### 3.1 Participants

During the study period, a total of 2,898 community residents aged 18–80 years were assessed for eligibility. Over the one-year follow-up period, 609 participants were lost to follow-up, resulting in an attrition rate of 21.0%. The reasons for loss to follow-up included changes in contact information, relocation outside the study communities, and withdrawal of consent. Consequently, 2,289 participants (531 young adults and 1,758 older adults) successfully completed the one-year follow-up and were included in the final longitudinal analysis, yielding an overall follow-up rate of 79.0% (Figure 1).

**Figure 1:**
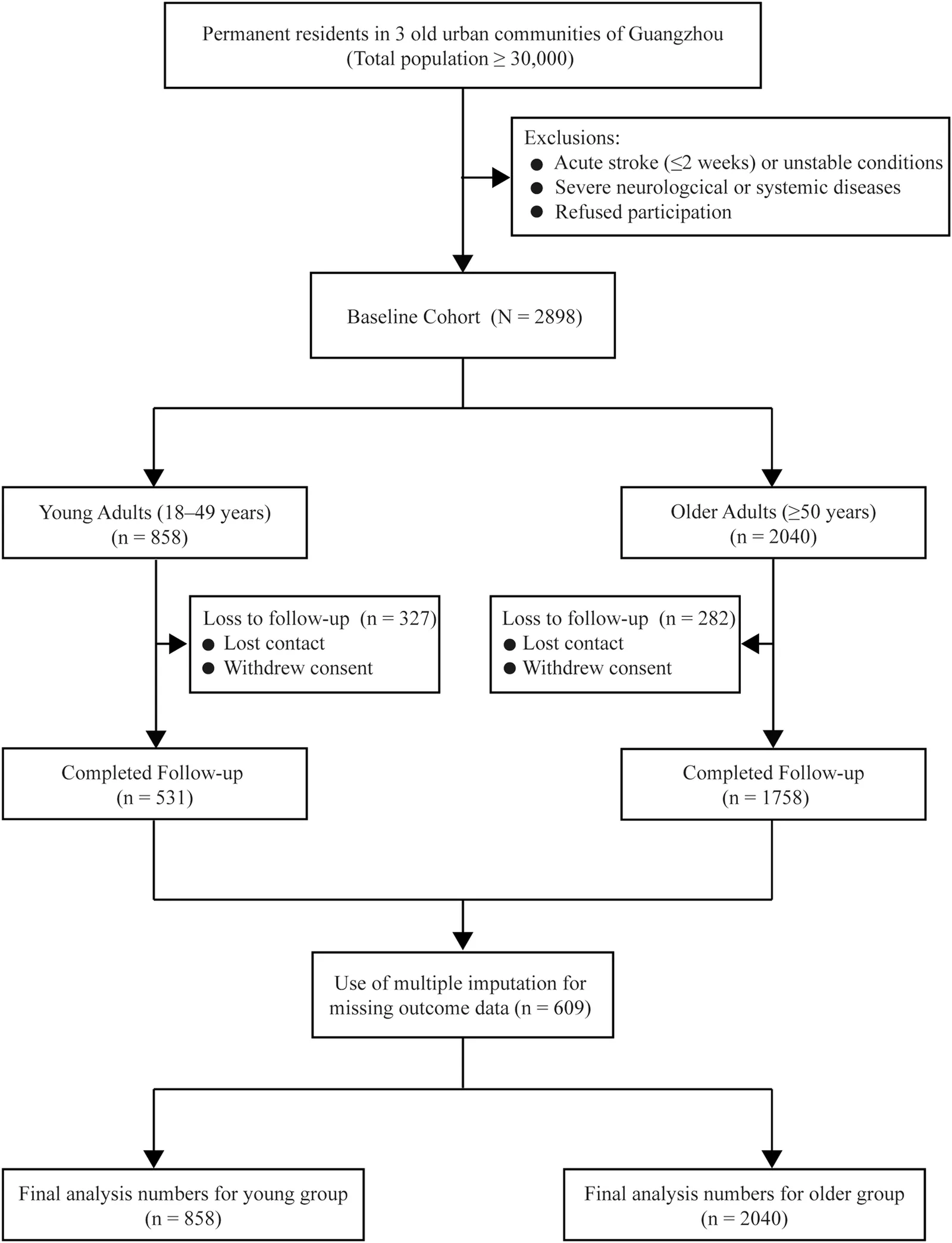
Flowchart illustrating a cohort study in Guangzhou with 2,898 participants divided into young adults (858) and older adults (2040).

Among them, 858 subjects (29.60%) were aged 18–49 years, with a mean age of 41.66 years and a male proportion of 41.84%. The remaining 2,040 subjects (70.39%) were aged ≥ 50 years, with a mean age of 61.65 years and a male proportion of 30.15%. Ultimately, 2,289 subjects completed the 1-year follow-up.

### 3.2 Baseline comparison of the two groups

Compared with the older adult cohort, young adults exhibited higher educational attainment and a significantly different occupational distribution. The older adult group had significantly higher prevalences of hypertension, dyslipidemia, diabetes mellitus, heart disease, anxiety or depression, cognitive impairment, hyperuricemia or gout, autoimmune diseases, endocrine and metabolic disorders, and a history of malignancy. Except for a higher prevalence of family histories of coronary artery disease and malignancy in the older group, family history profiles were largely comparable between the two cohorts.

Regarding lifestyle characteristics, the young adult group reported lower levels of physical activity, less time spent on domestic chores, and a lower frequency of interpersonal interactions. However, this group reported better sleep quality and was more likely to reside in cohabitation or collective living arrangements. Detailed baseline characteristics are summarized in Table 1.

**Table 1:** Comparison of demographic, clinical, and lifestyle characteristics of young and older adults in Guangzhou communities.

Characteristics	Categories	Young group（*n* = 858)	Older group（*n* = 2040)	*P* value
Number of health check-ups(%)	Once a year	711 (82.87%)	1934 (94.80%)	＜0.001
	Twice a year	143 (16.67%)	98 (4.80%)	
	Three times a year	4 (0.47%)	8 (0.39%)	
Gender	Female	499 (58.16%)	1425 (69.85%)	＜0.001
	Male	359 (41.84%)	615 (30.15%)	
Age(years)		41.66 ± 4.72	61.65 ± 7.37	
Educational attainment	Primary school and below	144 (16.78%)	788 (38.63%)	＜0.001
	Junior high school	180 (20.98%)	408 (20.00%)	
	Secondary vocational school/high school	360 (41.96%)	511 (25.05%)	
	Master's degree or above	174 (20.28%)	333 (16.32%)	
Occupation	Professional and technical personnel	76 (8.86%)	142 (6.96%)	＜0.001
	Individual business operator	33 (3.85%)	41 (2.01%)	
	Enterprise managers	69 (8.04%)	121 (5.93%)	
	Farmer	19 (2.21%)	168 (8.24%)	
	National civil servant	127 (14.80%)	565 (27.70%)	
	Student	0 (0.00%)	8 (0.39%)	
	Worker	111 (12.94%)	283 (13.87%)	
	Unemployed person	32 (3.73%)	122 (5.98%)	
	Active-duty military personnel	4 (0.47%)	6 (0.29%)	
	Staff	225 (26.22%)	254 (12.45%)	
	Freelancer	91 (10.61%)	91 (4.46%)	
	Others	71 (8.28%)	239 (11.72%)	
BMI		24.0 ± 7.0	23.9 ± 5.4	0.952
Previous medical history				
Hypertension	No	779 (90.79%)	1398 (68.53%)	＜0.001
	Yes	79 (9.21%)	642 (31.47%)	
Dyslipidemia	No	756 (88.11%)	1495 (73.28%)	＜0.001
	Yes	102 (11.89%)	545 (26.72%)	
Diabetes	No	840 (97.90%)	1825 (89.46%)	＜0.001
	Yes	18 (2.10%)	215 (10.54%)	
Heart disease	No	846 (98.60%)	1846 (90.49%)	＜0.001
	Yes	12 (1.40%)	194 (9.51%)	
Anxiety and depression	No	770 (89.74%)	1696 (83.14%)	＜0.001
	Yes	88 (10.26%)	344 (16.86%)	
Cognitive dysfunction	No	854 (99.53%)	1990 (97.55%)	＜0.001
	Yes	4 (0.47%)	50 (2.45%)	
Hyperuricemia or gout	No	775 (90.33%)	1729 (84.75%)	＜0.001
	Yes	83 (9.67%)	311 (15.25%)	
Autoimmune diseases	No	852 (99.30%)	1957 (95.93%)	＜0.001
	Yes	6 (0.70%)	83 (4.07%)	
Endocrine and metabolic diseases	No	833 (97.09%)	1900 (93.14%)	＜0.001
	Yes	25 (2.91%)	140 (6.86%)	
Tumor	No	845 (98.48%)	1951 (95.64%)	＜0.001
	Yes	13 (1.52%)	89 (4.36%)	
Genetic diseases	No	854 (99.53%)	2028 (99.41%)	0.79
	Yes	4 (0.47%)	12 (0.59%)	
Family history				
Stroke	No	744 (86.71%)	1722 (84.41%)	0.112
	Yes	114 (13.29%)	318 (15.59%)	
Coronary heart disease	No	787 (91.72%)	1796 (88.04%)	0.004
	Yes	71 (8.28%)	244 (11.96%)	
Hypertension	No	533 (62.12%)	1241 (60.83%)	0.516
	Yes	325 (37.88%)	799 (39.17%)	
Diabetes	No	713 (83.10%)	1752 (85.88%)	0.055
	Yes	145 (16.90%)	288 (14.12%)	
Hyperlipidemia	No	797 (92.89%)	1902 (93.24%)	0.738
	Yes	61 (7.11%)	138 (6.76%)	
Hyperuricemia or gout	No	803 (93.59%)	1923 (94.26%)	0.483
	Yes	55 (6.41%)	117 (5.74%)	
Tumor	No	789 (91.96%)	1800 (88.24%)	0.003
	Yes	69 (8.04%)	240 (11.76%)	
Endocrine and metabolic diseases	No	843 (98.25%)	2008 (98.43%)	0.727
	Yes	15 (1.75%)	32 (1.57%)	
Genetic diseases	No	853 (99.42%)	2032 (99.61%)	0.544
	Yes	5 (0.58%)	8 (0.39%)	
Lifestyle				
Smoking	No	734 (85.55%)	1750 (85.78%)	0.868
	Yes	124 (14.45%)	290 (14.22%)	
Drinking alcohol	No	688 (80.19%)	1660 (81.37%)	0.457
	Yes	170 (19.81%)	380 (18.63%)	
Exercise habit	People with insufficient exercise or light physical labor (exercise frequency less than 3 times per week and less than 30 min per session)	595 (69.35%)	1013 (49.66%)	＜0.001
	Regular exercise or heavy physical labor (including industrial and agricultural workers)	263 (30.65%)	1027 (50.34%)	
Manage the household	Don't do housework	175 (20.40%)	161 (7.89%)	＜0.001
	Do housework occasionally	97 (11.31%)	133 (6.52%)	
	Less than 30 min per day	90 (10.49%)	150 (7.35%)	
	30 min to 1 h per day	161 (18.76%)	317 (15.54%)	
	1 to 3 h per day	242 (28.21%)	824 (40.39%)	
	≥4 h per day	93 (10.84%)	455 (22.30%)	
Interpersonal communication	Basically none	276 (32.17%)	793 (38.87%)	＜0.001
	1-2 days a week	423 (49.30%)	724 (35.49%)	
	3 to 4 days a week	116 (13.52%)	345 (16.91%)	
	6 to 7 days a week	43 (5.01%)	178 (8.73%)	
Sleep habits				
Normal sleep	No	296 (34.50%)	945 (46.32%)	＜0.001
	Yes	562 (65.50%)	1095 (53.68%)	
Have difficulty falling asleep	No	791 (92.19%)	1607 (78.77%)	＜0.001
	Yes	67 (7.81%)	433 (21.23%)	
Wake up early	No	801 (93.36%)	1724 (84.51%)	＜0.001
	Yes	57 (6.64%)	316 (15.49%)	
It's difficult to fall asleep again after waking up	No	801 (93.36%)	1772 (86.86%)	＜0.001
	Yes	57 (6.64%)	268 (13.14%)	
Have many dreams	No	810 (94.41%)	1886 (92.45%)	0.059
	Yes	48 (5.59%)	154 (7.55%)	
Sleep duration	≤5 h per day	53 (6.18%)	416 (20.39%)	＜0.001
	5 to 8 h per day	608 (70.86%)	1378 (67.55%)	
	≥8 h per day	197 (22.96%)	246 (12.06%)	
Residence mode				
Live with one's spouse	No	397 (46.27%)	938 (45.98%)	0.886
	Yes	461 (53.73%)	1102 (54.02%)	
Live with children	No	486 (56.64%)	869 (42.60%)	＜0.001
	Yes	372 (43.36%)	1171 (57.40%)	
Live with parents	No	774 (90.21%)	1994 (97.75%)	＜0.001
	Yes	84(9.79%)	46(2.25%)	
Collective residence	No	688(80.19%)	1966(96.37%)	＜0.001
	Yes	170(19.81%)	74(3.63%)	
Living alone	No	824(96.04%)	1934(94.80%)	0.157
	Yes	34(3.96%)	106(5.20%)	

Effect estimates and *P*-values were calculated based on pooled data from multiple imputation (*N* = 2,898) to account for missing outcomes due to loss to follow-up.

### 3.3 Comparison of the 1-year prognosis

At the 1-year follow-up, modified Rankin Scale (mRS) scores were successfully obtained for all enrolled subjects; detailed clinical outcomes are summarized in Table 2. The results indicated that the young adult group had marginally worse functional outcomes than the older adult group, with a lower proportion of subjects achieving a favorable outcome (mRS 0–1: 60% vs. 65%).

**Table 2:** Comparison of mRS scores, ischemic stroke recurrence, and changes in essen stroke risk scores between young and older adults at the 1-year follow-up.

Outcome	Young group（*n* = 858)	Older group（*n* = 2040)	OR value (95% CI)	*P* value	Adjusted OR value(95% CI)	*P* value
Score of 0–1	515（60%）	1337（65.5%）	0.789（0.670–0.930）	<0.05	0.904（0.730–1.120）	0.335
Ischemic stroke recurrence	36 (4.20%)	118 (5.78%)	0.713（0.487–1.045）	0.083	1.135 (0.711–1.812)	0.459

	Young group	Older group	*β* coefficient (95% CI)		Adjusted *β* (95% CI)	
Essen difference	0（–2–0）	0（–1–1）	–0.016(−0.121–0.088)	<0.05	0.025(−0.089–0.138)	<0.05

Effect estimates and *P*-values were calculated based on pooled data from multiple imputation (*N* = 2,898) to account for missing outcomes due to loss to follow-up.

In the unadjusted analysis, the odds ratio (OR) for a more favorable mRS outcome in the young adult group, compared with the older adult group, was 0.789 (95% CI: 0.670–0.930, *P* < 0.05). However, after adjusting for potential confounders, this association was no longer statistically significant (adjusted OR: 0.904; 95% CI: 0.730–1.120; *P* = 0.355). The distribution of mRS scores for both age groups is illustrated in Figure 2.

**Figure 2:**
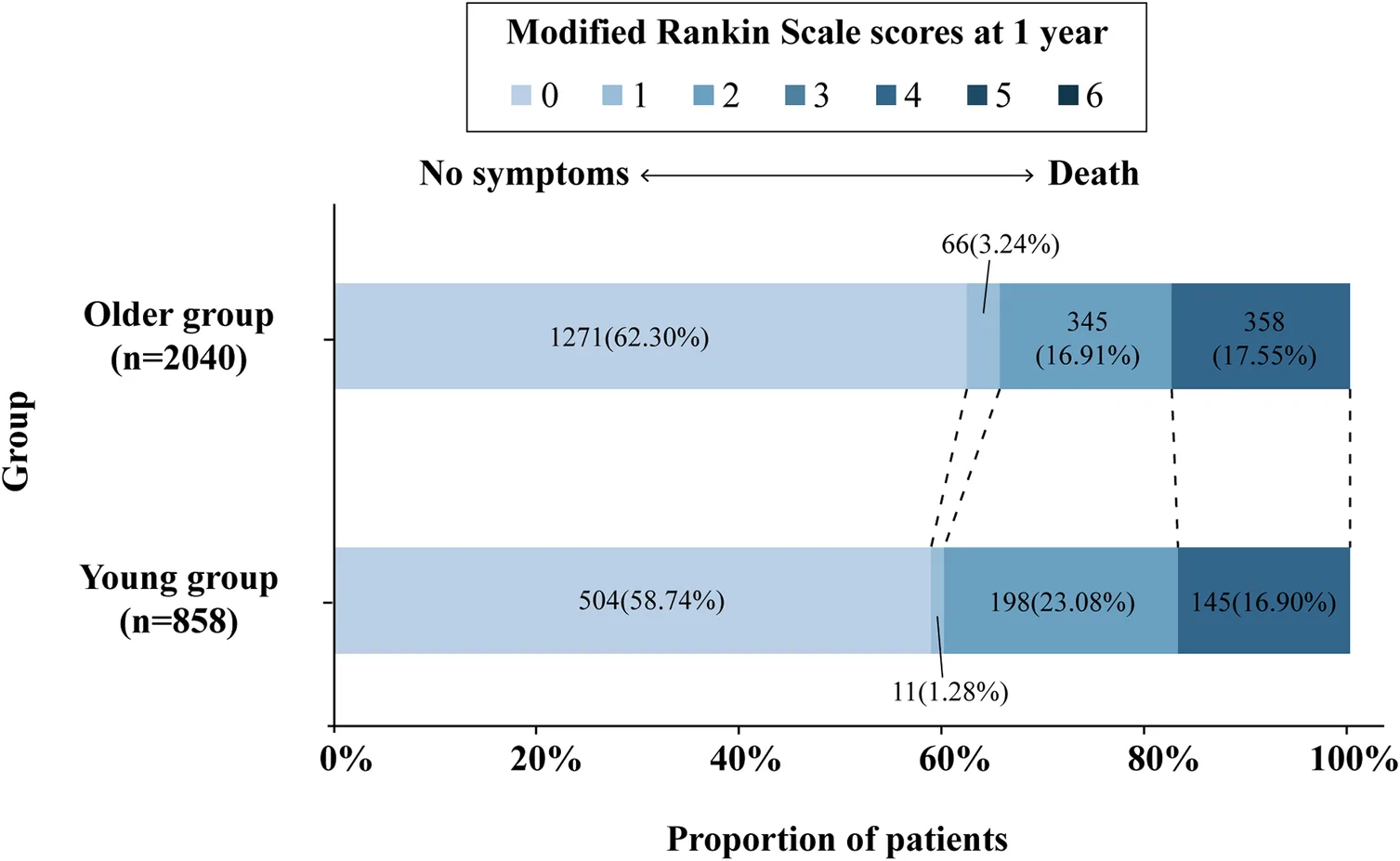
Distribution of Modified Rankin Scale (mRS) scores at 1-year follow-up in the young and older adult groups. Numbers in each colored block indicate the number of participants achieving that specific score at 1 year. A score of 0 indicates no symptoms, and a score of 6 indicates death. Data reflect the full baseline cohort (*N* = 2,898). Missing 1-year mRS scores were addressed via multiple imputation to account for participants lost to follow-up.

During the follow-up period, there were no statistically significant differences between the two groups regarding the overall recurrence of ischemic stroke. Nevertheless, the specific recurrence of ischemic stroke was lower in the young adult cohort than in the older adult cohort (4% vs. 6%). Conversely, the older group exhibited a more pronounced increase in Essen scores, suggesting a higher cumulative burden of risk factors for ischemic stroke.

## 4 Discussion

This study aimed to investigate the associations between dietary habits, physical activity, occupational characteristics, and other lifestyle-related factors and adverse clinical outcomes among young adults residing in the old urban area of Guangzhou. By comparing our findings with those of older cohorts, we sought to delineate the age-specific risk profile for stroke. Identifying these risk factors provides an evidence base for targeted preventive strategies centered on lifestyle modifications, behavioral interventions, and enhanced risk awareness, ultimately helping to preserve brain health and improve the quality of life for young community residents.

Our results demonstrated that in the unadjusted analysis, the young adult cohort (aged 18–49 years) exhibited a lower proportion of favorable functional outcomes at the 1-year follow-up compared to individuals aged 50 years and older. This suggests that the determinants of stroke prognosis in young adults may differ from those in older populations. Importantly, while the crude analysis showed a disparity in functional outcomes between the two age groups, this difference was attenuated and became statistically non-significant (adjusted OR 0.904, *p* = 0.355) after adjusting for lifestyle and baseline clinical factors. This attenuation suggests that these modifiable lifestyle factors may play an important role in mediating the worse functional outcomes initially observed in the unadjusted analysis.

Notably, a substantial proportion of the young participants were engaged in clerical or professional/technical occupations, which are typically characterized by prolonged sitting. Previous research has consistently established a significant association between sedentary behavior and elevated stroke risk, with this risk increasing progressively alongside sitting time (13). Furthermore, the young cohort reported lower levels of physical exercise, decreased participation in domestic chores, and fewer interpersonal interactions, collectively indicating a lower overall level of physical activity. A growing body of evidence underscores that physical inactivity and a sedentary lifestyle are closely linked to an increased risk of ischemic stroke; stroke prevalence is markedly higher among populations with less aerobic exercise and prolonged sedentary periods (14–17).

Conversely, older adults typically experience age-related organ decline and bear a heavier burden of chronic comorbidities. However, they generally possess greater health literacy and exhibit better self-management behaviors, which may mitigate their stroke risk and contribute to relatively better functional outcomes. Additionally, secondary stroke prevention strategies targeting older populations have been more extensively researched and implemented; moreover, multiple educational interventions have been proven effective in reducing the risk of stroke recurrence in this demographic.

Within our cohort, older subjects reported a higher prevalence of family histories of coronary heart disease (CHD) and malignancy. CHD and ischemic stroke frequently coexist and share common pathophysiological mechanisms, most notably atherosclerosis. Previous studies have shown that coronary artery stenosis is associated with increased recurrence of ischemic stroke, recurrent vascular events, and vascular death (18). In cancer patients, strokes are often driven by arterial or venous thromboembolism resulting from a hypercoagulable state, though tumor-related factors, infections, and therapeutic interventions may also be implicated. It is reported that approximately 5% to 10% of stroke patients have concurrent active malignancies (19). Although these findings seem to contradict the initially observed disparity in functional outcomes among the young cohort, it is crucial to note that even in the absence of a family history of CHD or cancer, young individuals may harbor other major stroke risk factors, including dyslipidemia, smoking, alcohol consumption, and obesity.

Moreover, prevalent unhealthy lifestyle behaviors among young adults—such as physical inactivity, high-salt and high-fat diets, and chronic sleep deprivation—may further elevate the risk of ischemic stroke. Sleep disorders, including obstructive sleep apnea (OSA) and insomnia, have been shown to significantly increase stroke risk and adversely affect post-stroke recovery, leading to poorer functional outcomes and higher recurrence rates (20). Our study did not specifically evaluate sleep apnea. Given that obesity, psychological stress, anxiety, and a higher male proportion were more common in the young cohort, the prevalence of sleep apnea in this demographic might be underestimated. Consequently, although young subjects self-reported better sleep quality, they may concurrently suffer from unrecognized sleep disorders, which could partially explain this initial disparity in functional outcomes. Nonetheless, this hypothesis requires validation in future studies incorporating objective sleep assessments.

Furthermore, young subjects were more inclined toward collective living arrangements, environments typically associated with higher ambient noise levels. Long-term exposure to environmental noise has been linked to an increased risk of severe stroke (21). Collective living environments may also restrict opportunities for physical activity, promote sedentary behavior, and increase the frequency of smoking and alcohol consumption, thereby elevating stroke risk.

Although worse functional status was observed in the young cohort in the unadjusted analysis, this difference lost statistical significance after adjusting for confounding variables. This finding implies that established lifestyle-related risk factors may contribute to both the onset of stroke and its poor prognosis in young adults. The initially poorer functional outcomes in the young cohort, compared to older adults, might also be partially attributable to lower compliance with preventive and rehabilitative interventions during the follow-up period, whereas older populations might exhibit higher engagement in health self-management (22, 23). Even so, it is reasonable to hypothesize that targeted management of these modifiable risk factors could significantly reduce stroke recurrence and improve outcomes in young adults. Consistent with this interpretation, while ischemic stroke recurrence rates were comparable between the two groups, the young group demonstrated a smaller magnitude of change in the ESRS, likely reflecting their comparatively better physiological reserve at study enrollment.

This study has several limitations that warrant acknowledgment. First, the relatively small sample size and the recruitment of participants from communities within a single urban district may restrict the generalizability of our findings. Additionally, potential selection bias related to informed consent and loss to follow-up cannot be definitively ruled out. Second, our reliance on structured questionnaires for assessing lifestyle factors (e.g., physical activity and sleep quality) and self-reported, physician-diagnosed histories for baseline comorbidities introduces potential recall bias. Although these standardized epidemiological methods are well-accepted for large-scale community cohorts, the lack of objective verification—such as wearable monitoring devices, polysomnography (particularly for obstructive sleep apnea), or direct medical chart reviews—limits the granularity of our data. Third, due to the observational nature of this cohort, strict causal relationships between specific modern lifestyle factors and worse functional outcomes (mRS scores) cannot be definitively established. Furthermore, while the attenuation of the age effect after adjusting for lifestyle factors strongly suggests a mediating role of these contemporary habits, we did not perform a formal statistical mediation analysis (e.g., path analysis) to quantify this effect. Fourth, owing to the community-based design of our data collection, we lacked access to detailed acute-phase clinical characteristics from the participants’ initial hospitalizations, such as baseline functional status, specific stroke subtype, initial stroke severity, and the exact time elapsed since the index stroke. The inability to adjust for these critical stroke-related variables may introduce residual confounding. Finally, the relatively short follow-up duration makes it difficult to ascertain whether the observed associations remain stable over time. Future large-scale, multicenter prospective studies—employing objective monitoring tools, extended follow-up periods, and formal mediation models—are necessary to address these limitations, confirm these mechanistic pathways, and optimize secondary prevention strategies for young stroke patients.

## 5 Conclusion

In conclusion, while unadjusted analyses initially suggested a lower proportion of favorable functional outcomes among young adults (aged 18–49 years) compared to older adults (aged ≥50 years), this disparity was attenuated after comprehensive adjustment. Additionally, ischemic stroke recurrence rates were comparable between the two age groups. These findings suggest that the functional recovery of young stroke patients may be importantly mediated by their distinct, modifiable lifestyle behaviors and baseline risk factor profiles. Future research is warranted to elucidate the underlying mechanisms governing stroke onset and post-stroke recovery in this population. Ultimately, tailoring age-appropriate preventive interventions to address these modifiable lifestyle factors may play a pivotal role in reducing long-term stroke burden and optimizing functional outcomes.

## Data Availability

The raw data supporting the conclusions of this article will be made available by the authors, without undue reservation.
